# Arterial-colonic fistula secondary to colonic stent erosion into the left external iliac artery

**DOI:** 10.1093/jscr/rjac615

**Published:** 2023-01-10

**Authors:** Jacquelyn Dillon, Alexandra N Mills, Kate R Pawloski, Nicholas Scribetta, Alexander Greenstein

**Affiliations:** Department of Surgery, Icahn School of Medicine at Mount Sinai, New York, NY, USA; Department of Surgery, Icahn School of Medicine at Mount Sinai, New York, NY, USA; Department of Surgery, Icahn School of Medicine at Mount Sinai, New York, NY, USA; Department of Pathology, Molecular and Cell-Based Medicine, Icahn School of Medicine at Mount Sinai, New York, NY, USA; Department of Surgery, Icahn School of Medicine at Mount Sinai, New York, NY, USA

**Keywords:** self-expandable metal stent, colonic stent, fistula

## Abstract

Self-expandable metal stent (SEMS) are widely utilized as a bridge to surgical intervention and for palliative treatment of malignant bowel obstructions. The risk of complications associated with SEMS is low in well-selected patients. Stent erosion is a rare but serious adverse event that is associated with high morbidity and mortality. Here, we report the case of a 74-year-old patient with a colonic obstruction secondary to a pelvic mass that was treated with SEMS and radiotherapy, who developed a partial thickness stent erosion and recurrent hematochezia 6 years after placement. Endoscopic retrieval was not technically feasible. During attempted surgical resection, massive hemorrhage occurred from a colonic-arterial fistula to the left external iliac artery resulting in death. While SEMS remain an effective, minimally invasive approach for the management of bowel obstructions, prolonged *in-situ* lifetime may confer an increased risk of serious adverse events including erosion and fistula formation.

## INTRODUCTION

Intestinal obstructions are commonly caused by adhesions, hernias, strictures and malignancy. Self-expanding metal stents (SEMS) are increasingly utilized for palliative treatment of malignant colonic obstructions and may be used as a bridge to surgery in patients undergoing medical and nutritional optimization [1]. SEMS are associated with shorter hospital stays, decreased costs, lower rates of stoma formation, reduced morbidity and improved quality of life compared with emergency surgical intervention in appropriately selected patients [1]. While SEMs may be used to treat benign strictures, i.e. diverticulitis and inflammatory bowel disease [2–5], they are not considered first-line therapy because of the risk of serious complications associated with longer *in-situ* lifetime including stent erosion and fistula formation [6, 7].

Long-term complication rates associated with SEMS are not well-defined. For patients with metastatic colorectal cancer, improved systemic therapy may extend treatment duration and *in-situ* stent lifetime. Whether the risk of stent-related complications is increased by the receipt of abdominopelvic radiation is unknown. Here, we report the case of a 74-year-old patient with a malignant colonic obstruction secondary to a pelvic mass that was treated with SEMS and radiotherapy with a stent *in-situ* time of 78 months, who developed a colonic-arterial fistula to the left external iliac artery. To our knowledge, this is the first reported case of colonic stent erosion and fistulization leading to hemorrhage and death.

## CASE REPORT

A 74-year-old woman presented to the emergency department with multiple self-limited episodes of hematochezia. Six years prior to presentation, she had undergone placement of a left colonic WallFlex stent for a large bowel obstruction. Her surgical history included a total abdominal hysterectomy and bilateral salpingo-oophorectomy for endometrial cancer. She experienced a recurrence 2 years later involving the bladder, vagina and sigmoid colon for which she had undergone debulking, segmental colectomy with primary anastomosis, chemotherapy and external beam pelvic radiation. Two years later, she developed a high-grade large bowel obstruction secondary to a benign stricture at the colorectal anastomosis. A 12 cm × 25 mm uncovered WallFlex colonic stent provided temporary relief of her obstructive symptoms. One year later she experienced recurrent pain, obstipation and intermittent, self-limited episodes of hematochezia. Colonoscopy revealed ulcerated mucosa within and just proximal to the stent with luminal narrowing; pathology showed mildly active, nonspecific colitis. The patient elected to undergo surgical intervention to treat her obstruction. Because of extensive adhesions precluding safe dissection, an end colostomy was matured, and the stent was left *in situ* within long a Hartmann’s stump.

**Figure 1:**
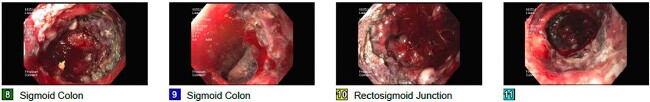
Flexible sigmoidoscopy 72 months following stent placement.

**Figure 2:**
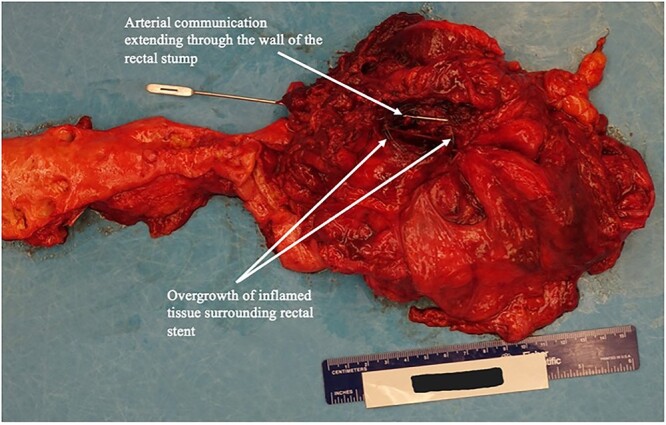
Autopsy image rectal stump and of arterio-rectal communication with left EIA.

**Figure 3:**
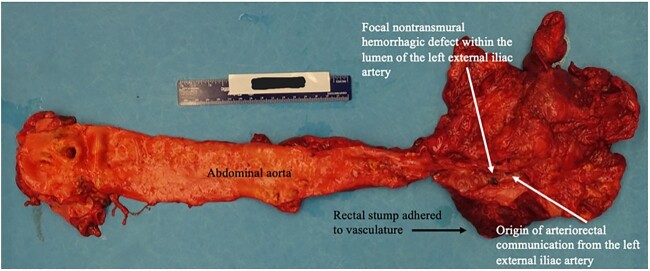
Autopsy image of abdominal aorta including site of arterio-rectal communication with left EIA.

Five years later, she continued to experience intermittent lower GI bleeding requiring transfusions, leading to her current presentation. Flexible sigmoidoscopy demonstrated partial stent erosion into the mucosa with tissue ingrowth and severe friability within the Hartmann’s stump (Fig. 1). The stent could not be removed endoscopically. She was counseled regarding treatment options and risks associated with further attempts at removal. The patient strongly desired additional intervention and was taken to the operating room following optimization with a plan to excise the rectal stump and stent. Upon exploration, an inflamed Hartmann’s stump was found to be densely adherent to the pelvic sidewall. Arterial hemorrhage was encountered soon after careful attempt to bluntly dissect the stump. Temporary control was achieved with pressure and vascular surgery was consulted. Brisk hemorrhage from the left external iliac artery (EIA) was noted upon further mobilization of the stump. The vessel was dissected proximally in an effort to ligate the left EIA and perform a femoral–femoral bypass. Despite resuscitative efforts, the patient went into cardiac arrest and expired after multiple rounds of ACLS. An autopsy revealed full-thickness erosion of the stent with metallic mesh wires embedded throughout the exposed colonic mucosa (Fig. 2) and transmural erosion of the stent into the left EIA with hemorrhage (Fig. 3).

## DISCUSSION

SEMs are increasingly used to treat malignant bowel obstructions and benign strictures in select patients. Potential complications include migration, erosion, perforation, bleeding, need for additional procedures if decompression is inadequate, and rarely mortality. The risk of stent erosion causing intestinal perforation is reported at 4.8–6.4% [2, 8], with an associated mortality risk of 16% [9]. Limited data suggest that perforation is associated with the depth of tumor invasion and endoscopic balloon dilation after stent placement [1]. Hemodynamically significant bleeding secondary to erosion has not previously been described. We report the first case, to our knowledge, of full-thickness stent erosion with resultant arterial-colonic fistula and hemorrhage.

Previous case reports have described entero-enteric fistula formation due to stent erosion [10–12]. Examples include a patient with Crohn’s disease with a terminal ileal stricture complicated by perforation at the ileocecal junction and ileo-sigmoid fistula formation after stent placement [12]. Another report describes erosion of a WallFlex stent placed for malignant stricture at the anal verge; the patient was found to have a full-thickness stent erosion through the colon wall into the distal ileum. Exploration revealed adherence of the stent, colon and distal ileum to the iliac bifurcation on the posterior pelvic wall [10]. Mechanistically, erosion may occur following a localized inflammatory reaction leading to fistula formation in patients with friable surrounding tissue planes. In select circumstances, the degree of luminal occlusion may be exacerbated by prior radiation. Careful patient selection vis-à-vis anatomic location of the obstruction, degree of occlusion and tissue quality may help to reduce complications. Rectal stents are associated with higher complications rates because of greater mechanical stress and are poorly tolerated.

Colonic stents remain an excellent option to palliate malignant obstructions. In this setting, patients frequently experience symptomatic relief, have shorter hospital stays, lower risk of adverse events and lower hospital mortality compared with those undergoing palliative surgery [1, 13, 14]. A prior study reported viable stent function without complication after 89 months in a patient treated with a palliative stent [15]. Caution is warranted in patients who have longer life expectancy given limited data regarding the long-term safety.

## CONCLUSIONS

Overall survival among patients with advanced colorectal and gynecologic malignancies is improving with advancements in systemic therapy. In patients with unresectable disease, palliative stents may improve quality of life and reduce surgical risks. Patients should be counseled on the rare but serious risks including death. Close follow-up is warranted, particularly if the stent remains *in situ* > 12 months.

## AUTHORS’ CONTRIBUTIONS

All authors contributed to the writing, editing and approval of the final manuscript.

## CONFLICT OF INTEREST STATEMENT

None declared

## FUNDING

None.

## DATA AVAILABILITY

Not applicable.
